# The complex network of p300/CBP regulation: Interactions, posttranslational modifications, and therapeutic implications

**DOI:** 10.1016/j.jbc.2025.110715

**Published:** 2025-09-15

**Authors:** Ebru Yazıcı, Justyna McIntyre

**Affiliations:** Institute of Biochemistry and Biophysics, Polish Academy of Sciences, Warsaw, Poland

**Keywords:** p300, CBP, acetyltransferases, acetylation, ubiquitination

## Abstract

Two closely related acetyltransferases, p300 and its paralog CBP, are important regulators of gene expression and protein modulators in higher eukaryotes, influencing a wide array of cellular processes, including cell division, growth, DNA replication and repair, and apoptosis. The broad cellular impact is underscored by p300/CBP's capacity to interact with hundreds of proteins through various domains and the capability to acetylate numerous substrates and ubiquitinate selected targets. This intricate network of interactions and modifications highlights the essential role of p300/CBP in orchestrating cellular responses to pathological and physiological stimuli, thereby necessitating precise regulatory mechanisms to maintain their activity and substrate specificity. The regulation of p300/CBP is primarily governed by protein interactions and posttranslational modifications, including acetylation and ubiquitination, with autoregulation serving as a vital component in sustaining their enzymatic functions. The significance of tightly controlled p300/CBP activity is further emphasized by its association with various diseases, including Rubinstein–Taybi syndrome, Menke–Hennekam syndrome, and numerous cancers. Furthermore, the potential of p300/CBP as a therapeutic target has sparked interest in developing specific inhibitors. This review aims to elucidate the complex regulatory mechanisms of p300/CBP, focusing on posttranslational modifications, intermolecular interactions, and their implications in disease. A comprehensive understanding of the molecular foundations of p300/CBP regulation is essential for unraveling their roles in cellular processes and advancing targeted therapeutic strategies.

N-ε-acetylation or "acetylation" is a pivotal and reversible posttranslational protein modification significantly influencing protein function and cellular processes. Two principal classes of enzymes orchestrate this modification: lysine acetyltransferases, which catalyze the transfer of an acetyl moiety to a lysine residue; and lysine deacetylases, which facilitate their removal [reviewed in (1)]. One of the most important acetyltransferases is the E1A binding protein p300, encoded by the *EP300* gene and its paralog CBP (the cyclic AMP response element-binding protein (CREB)-binding protein, encoded by *CREBBP* gene), collectively referred to as p300/CBP. These proteins are primarily known for their essential role in histone acetylation, which modulates transcriptional regulation through chromatin remodeling. However, beyond histones, p300 and CBP are also involved in acetylating a diverse array of transcription factors and coactivators, thereby influencing their activity and functionality [(2), reviewed by (3)]. Recent advancements in acetylome analysis have revealed approximately 21,000 acetylation sites regulated by p300/CBP across more than 5000 proteins (4). Additionally, p300 possesses a noncanonical RING domain typical of ubiquitin ligases, allowing it to ubiquitinate certain proteins, including tumor suppressor p53 (5, 6). Remarkably, this ubiquitin ligase activity of p300/CBP is exclusively cytoplasmic and leads to p53 destabilization, while p300/CBP-dependent nuclear acetylation activates p53 (6). Besides regulating proteins by posttranslational modifications (PTMs), p300 has an extensive interaction network, involving over 400 proteins (7). Together, by protein modifications and protein–protein interactions, p300 impacts a myriad of cellular mechanisms and pathways that, in addition to transcription, affect cell cycle progression, DNA replication, DNA repair, nuclear import, and others [reviewed by (8)]. Thus, p300 and CBP regulate numerous fundamental biological processes, including proliferation, differentiation, embryogenesis, cell development, tumorigenesis, as well as immune response regulation and apoptosis (9, 10, 11, 12).

In humans, the *EP300* gene is located on chromosome 22 (22q13) and encodes a 300 kDa protein belonging to the lysine acetyltransferase 3 family (13). p300 has nuclear and cytoplasmic cellular localizations and is ubiquitously expressed across human tissues, with particularly high levels found in bone marrow (https://www.proteinatlas.org/ENSG00000100393-EP300). Unlike prokaryotes and lower eukaryotes, which lack homologs of p300, multicellular organisms possess either p300 or CBP (3, 8, 14, 15). p300 and CBP are paralogs that diverged as a consequence of gene duplication over 450 million years ago (16). As p300 and CBP share an overall 58% sequence identity, 86% amino acid identity of the catalytic region, and even higher for some other domains, they frequently overlap in function and are therefore often referred to as p300/CBP (16, 17, 18). Despite their functional overlap, p300 and CBP are not entirely redundant, and some unique functions have been identified (19, 20, 21). p300 and CBP show distinct roles in processes like muscle differentiation, vascular smooth muscle cell phenotypic switching, embryonic heart development, or fat remodeling regulation (22, 23, 24). Despite the high overlap in the transcription factors targeted by p300 and CBP, some were found to be specific for one or the other (25). Additionally, the two enzymes differ in the specificity of histone acetylation. While CBP greatly favors H3K18 acetylation over any other H3 or H4 acetylation, p300 specificity toward different sites (K9, K14, K18, and K23 of H3) is more balanced (26). Moreover, in mouse embryonic stem cells, p300, but not CBP, contributes to H3K27ac at regulatory regions of the genome (27). Generally, in contrast to most results of *in vitro* studies, the diversity of the two acetyltransferases is clearer *in vivo*. It is underlined by the fact that *EP300* or *CREBBP* homozygotes die at an early stage of embryonic development, and double heterozygous mice die before birth. Additionally, CBP is unable to rescue the growth defect in p300-deficient carcinoma cells, and *vice versa*. Additionally, various diseases are caused by defects in a specific acetyltransferase (28). Mutations in CBP are dominantly associated with Rubinstein–Taybi syndrome (RSTS) (29, 30), whereas defects in p300 are related to particular types of cancer (31, 32). Moreover, mutations, defects, dysregulation, and functional inactivation of p300/CBP are associated with various other disorders, including Menke–Hennekam syndrome (MHS) (33), neurodegenerative diseases, such as Huntington (34), bulbar muscular atrophy, heart malfunction, diabetes mellitus, as well as different types of tumors (8).

Given the extensive regulatory roles of p300/CBP in cellular mechanisms and the implications of its dysregulation in numerous diseases, understanding the regulation of p300/CBP itself is of paramount importance. While numerous reviews have addressed the p300/CBP-dependent control of various cellular processes (35, 36, 37, 38, 39, 40), there remains a notable gap in the literature focusing specifically on the regulation of p300 itself.

This review summarizes the multifaceted regulation of p300/CBP, concentrating on PTMs, intramolecular and intermolecular interactions, and disease implications. Understanding the molecular basis of p300/CBP regulation is crucial for deciphering various cellular processes and elucidating the role of p300/CBP in disease, as well as for developing targeted therapeutic strategies.

## The structure of the p300

**Figure 1:**
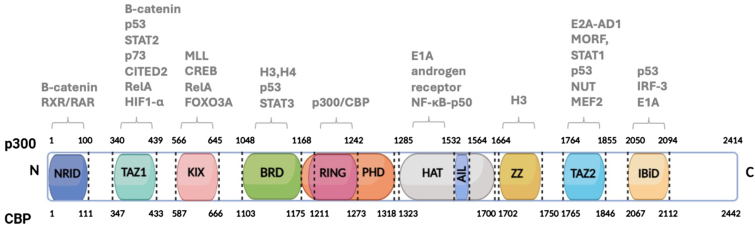
**Domain organization of p300/CBP.** p300/CBP contains multiple functional domains: the N-terminal nuclear receptor interaction domain (NRID), transcriptional adapter zinc finger 1 (TAZ1), kinase-inducible domain of CREB-interaction domain (KIX), bromodomain (BRD), RING domain, plant homeodomain (PHD), p300 histone acetyltransferase (HAT) domain, autoinhibitory loop (AIL), ZZ-type zinc finger domain (ZZ), TAZ2 domain, and N-terminal interferon-binding domain (IBiD). The coordinates of domains in p300 are marked in the *upper line* and CBP in the *lower line*. The domain sizes and distances between domains in the diagram do not reflect the real perspective. Intrinsically disordered regions are marked in *white*. Interacting proteins for each domain are listed above the diagram. CBP, CREB-binding protein.

In addition to its histone acetyltransferase (HAT) domain, which facilitates the transfer of acetyl groups to lysine residues on substrate proteins, p300 also harbors a second catalytic domain known as RING. Despite being noncanonical, the RING domain functions as an E3 ubiquitin ligase. These two enzymatic domains are flanked by a bromodomain (BRD) and a plant homeodomain (PHD), which, together with HAT and RING, constitute a centrally positioned catalytic core spanning residues 1048 to 1664 (Fig. 1) (41). The core region of p300/CBP is largely conserved among multicellular species (41).

Beyond its catalytic domains, p300 comprises multiple evolutionarily conserved, noncatalytic regions characterized by distinct protein–protein interaction motifs. These include the nuclear receptor interaction domain (NRID), two transcriptional adapter zinc-binding domains (TAZ1 and TAZ2), the kinase-inducible domain of CREB-interacting domain (KIX), a ZZ-type zinc finger (ZZ), and an interferon-binding domain (IBiD). Additionally, three cysteine/histidine-rich regions (CH1, CH2, and CH3) were founded within the TAZ1, RING-PHD, and ZZ-TAZ2 domains, respectively (Fig. 1). A striking feature of p300 is the presence of extensive intrinsically disordered regions (IDRs), comprising nearly 60% of its sequence, located between its structured domains and the catalytic core. Emerging evidence indicates that, initially believed to serve only as flexible linkers, these regions are now suggested to play regulatory roles beyond mere structural connectivity (42, 43). The functions and interacting partners of various p300/CBP domains are summarized in Table 1.

**Table 1:** p300/CBP domains and functions

Domain	Interacting proteins	Function	References
NRID	β-Catenin, retinoic acid receptors (RXR/RAR)	• Involved in mediating protein–protein interactions.	(44, 207)
TAZ1	β-Catenin, p53, STAT2, p73, hypoxia-inducible factor-1 alpha (HIF-1α), CITED2, RelA, STAT2	• Stabilizes transcriptional machinery. • Plays a crucial role in activating transcription in response to stress or immune signals.	(45, 46, 47, 48, 49, 50, 208, 209, 210)
KIX	c-Myb and mixed lineage leukemia protein (MLL), Forkhead box class O 3a (FOXO3a), c-Jun, E2A, RelA	• Serves as the primary interaction site for hematopoietic transcription factors. • Acts as a docking platform for transcription factors involved in cell growth, differentiation, and stress responses.	(50, 59, 211, 212, 213, 214, 215)
Bromodomain	Acetylated histones (H3, H4), acetylated p53, STAT3	• Recognizes acetylated lysine residues. • Promotes chromatin remodeling and transcriptional activation.	(72, 216, 217, 218, 219)
RING	Intramolecular with the HAT domain	• Catalyzes protein ubiquitination (E3 ligase). • Functions as a regulator of HAT activity	(41, 98)
PHD	Chromatin	• Involved in transcriptional regulation by reading chromatin marks.	(81, 218, 220)
HAT	Histones (H3, H4), E1A, androgen receptor, NF-κB-p50	• Acetylates histones and non-histone proteins, including itself. • Regulates chromatin structure and transcription.	(96, 221)
ZZ	Histone H3 tail	• Stimulates *cis* acetylation of H3K27 and H3K18. • May serve as a new epigenetic reader.	(67)
TAZ2	The E-protein transcription factors (E2A)—activation domains (ADs)1, p53, nuclear protein in testis (NUT), MORF, myocyte enhancer factor 2 (MEF2), β-catenin, STAT1	• Enhances and activates histone acetylation. • Promotes protein–protein interactions.	(49, 52, 53, 54, 55, 56, 156, 208, 222)
IBiD	p53, IRF-3, E1A	• Mediates the protein–protein interactions. • Serves as a key component for integrating cellular signals.	(89, 91, 208)

CBP, CREB-binding protein; HAT, histone acetyltransferase; IBiD, interferon-binding domain; KIX, kinase-inducible domain-of CREB-interacting domain; PHD, plant homeodomain; TAZ, transcriptional adapter zinc-binding domain; ZZ, ZZ-type zinc finger.

### Nuclear receptor interaction domain (NRID)

As the name indicates, the NRID domain plays a role in associating nuclear receptors. However, the N-terminal region of p300/CBP, including NRID, can also interact with other proteins, such as β-catenin (44). The NRID domains of p300 and CBP share the lowest sequence identity (63%) and are the unique targets for developing compounds that distinguish between CBP and p300 (16).

### TAZ domains

The p300/CBP structure contains two TAZ domains: TAZ1, located within the CH1, and TAZ2, located in the CH3 region. The principal function of the TAZ domains seems to be protein recognition, and over 30 distinct transcription factors connect with p300/CBP *via* TAZ domains (45). Even though the TAZ1 and TAZ2 domains have similar sequences and adopt similar folds of a bundle of four helices stabilized by three zinc atoms, they connect to distinct transcription factor subsets. TAZ1 can interact with hypoxia-inducible factor-1α (46), β-catenin (47), CITED2 (48), STAT2 (49), a prominent NF-κB family member RelA (50), p53 (51), and TAZ2 with p53 (52, 53), β-catenin (47), MORF (54), STAT1 (49), E2A-AD1 (55), and MEF2 (56).

Both TAZ domains play important regulatory roles in coordinating transcription complexes. While transcription factors often compete for binding to TAZ1, TAZ2 facilitates acetylation-dependent mechanisms by positioning transcription factors in proximity to the HAT domain [reviewed in (57)]. Additionally, TAZ1 plays an important role in the efficient recruitment of p300/CBP to hypoxia-inducible factor target genes, accounting for up to 50% of hypoxia-responsive gene expression (58).

### KIX domain

The KIX domain of p300/CBP is a highly conserved and independently folded three-helix bundle that serves as a critical hub for protein–protein interactions, essential for gene regulation. It interacts with IDRs of transcription factors, stabilizing their structure upon binding, which is critical for effective transcriptional activation [reviewed in (59)]. KIX interacts with many transcriptional factors involved in hematopoietic differentiation, such as CREB, Myb, MLL, c-Jun, E2A, and FOXO3 (see Table 1). Recent research indicates that the Myb–KIX interaction plays a significant role in a subset of acute myeloid leukemia (60). Additionally, along with TAZ1, TAZ2, and IBiD, KIX is an activator-binding domain, and all four domains share binding partners [reviewed (57)]. The KIX structural motif has been identified in multiple proteins that perform diverse roles (61, 62, 63). In addition to its coactivator role, the KIX domain also modulates the protumorigenic function of p300. Importantly, research by Kim *et al.* demonstrates that disrupting KIX-mediated interactions inhibit small cell lung cancer progression and reduce tumor cell proliferation *in vivo* (64). This highlights the potential of targeting KIX as a therapeutic strategy, not only for acute myeloid leukemia but also for other malignancies where its dysregulation may contribute to tumor progression. So, targeting the KIX domain could lead to innovative treatment options that enhance patient outcomes by disrupting the pathways involved in tumorigenesis and promoting cancer cell apoptosis.

These findings emphasize the important role of the KIX domain in p300/CBP-mediated transcriptional regulation and its broader implications in cancer biology.

### Bromodomain

The BRD represents a large family of evolutionarily conserved protein modules found in a wide range of chromatin-associated proteins, almost all known HATs, and methyltransferases (65). These domains, besides playing a central role in mediating protein interactions, can recognize and bind acetylated substrates, which underscores their regulatory role (66). BRD of the p300 is essential for identifying acetylated histones, particularly H3 and H4 (H3K27ac, H3K18ac, and H4K12ac, N-terminal tail of H4, respectively), but also N-terminal tail of H2A.Z, an evolutionary conserved variant of H2A, and acts as a "reader" of these epigenetic histone marks to control the structure of chromatin (67, 68). The p300 BRD, beyond interacting with histones, has also non-histone protein partners including transcription factors (*e.g.*, acetylated myogenic factor MyoD, essential in transactivating muscle-specific promoters (69)), nuclear receptors, and enzymes, playing central roles in the positive regulation of transcription [reviewed in (70)]. However, some other protein partners (*e.g.*, C-terminal binding protein), by binding to BRD and recruiting p300, can act as a transcriptional corepressor (71). Consequently, BRD acts as an anchoring module, facilitating p300 association with chromatin to promote histone acetylation, chromatin remodeling, and transcriptional activation (65, 72, 73, 74). p300 BRD has been suggested to preferentially bind multiacetylated peptides (75). By binding to the acetyl-lysine site of p300, the BRD acts as its autoregulator and, together with the PHD, RING, and HAT domains, forms an organized regulatory unit, in which the RING domain is located near the substrate binding site of HAT (41).

Dysregulation of BRD activity has been implicated in various cancer types, highlighting its importance in maintaining cellular homeostasis. Additionally, the expression level of p300/CBP BRD correlates with cancer prognosis, making it a potential target for therapeutic inhibitors (76, 77, 78).

### The RING domain

RING domains are characteristic of many E3 ubiquitin ligases involved in an enzymatic (E1-E2-E3) cascade leading to specific protein ubiquitination. RING E3 enzymes mediate the transfer of ubiquitin directly from E2∼ubiquitin to a substrate protein (79). In contrast to most E3 RINGs, which coordinate two Zn^2+^ ions, the p300 RING binds only one zinc ion, while the second metal-binding site is stabilized through hydrophobic interactions (41). This unique configuration highlights the distinct functional characteristics of the p300 RING domain. Additionally, the RING domain plays a crucial role in the regulation of HAT activity, further emphasizing its significance in cellular processes (more in chapter 3.1.3).

### PHD finger

The PHD finger domains are found in approximately 300 predominantly nuclear proteins where they play a role in transcriptional regulation within a chromatin context (80). The PHD finger functions by coordinating zinc ions, and when zinc is chelated, it can destabilize the finger’s structure, thereby impairing the activity of the adjacent acetyltransferase domain. The functional significance of the PHD finger is underscored by the fact that mutations in this domain, which affect CBP/HAT activity, have been associated with RSTS (81, 82).

### The HAT domain and autoinhibitory loop

The primary function of the HAT domain is to modulate chromatin structure through the acetylation of histones and associated proteins, thereby "relaxing" the chromatin and enabling the transcriptional activation of nearby genes. Following histone acetylation, the adjacent BRD recognizes and binds to the acetylated histone tails, which is a critical step in mediating chromatin dynamics (17, 83). The HAT domains of p300 and CBP share over 90% sequence similarity, reflecting their highly conserved function (84). However, distinct structural features differentiate p300 from other HATs. The acetyl-CoA binding site in p300 is more concealed, and the substrate binding site is deeper, with a negatively charged surface, in contrast to the shallow, apolar pockets found in other HATs (85). Notably, in contrast to most acetyltransferases, the p300 HAT domain does not form a stable ternary complex with substrates. Instead, it has been proposed that the lysine residue of the substrate peptide traverses a tunnel within p300, where it transiently engages with the acetyl group in a "hit-and-run" (Theorell–Chance) catalytic mechanism (84, 85). Based on the mutational analysis, some key residues, including E1505 and D1628, were pointed out to be critical for the catalytic process. Specifically, the D1628R mutation leads to a significant reduction in p300 HAT activity, further underscoring the importance of these residues in the enzyme's function (86).

Within the HAT domain, a lysine-rich and IDR named autoinhibitory loop (AIL, 1532–1564 aa in p300 and 1556–1618 aa in CBP in human cells), plays an important role in the regulation of the HAT activity.

### Zinc finger domain

The ZZ domain derives its name from the two Zn^2+^ ions it can bind (87). The proteins containing ZZ domains vary in function and domain composition, but often possess additional zinc-binding motifs (in the case of p300/CBP, it is TAZ) (88). Those proteins can be divided into four main functional categories: chromatin modifiers, cytoskeletal modifiers, ubiquitin-binding or conjugating proteins, and membrane receptor or ion channel modifiers, and p300/CBP belongs to the first group. The ZZ domain of p300/CBP recognizes and binds the histone H3 tail and promotes histone H3K27 and H3K18 acetylation (67).

### IBiD

The C terminally located IBiD contains a nuclear coactivator binding domain and a glutamine-rich domain followed by a proline-containing PxP motif (3). Despite being a small domain, IBiD is capable of folding independently, without the need for stabilization by metal ions or a disulfide bond. It belongs to a class of proteins referred to as minidomains. A stable, correctly folded IBiD structure is required for mediating the protein–protein interactions, which are crucial for the recruitment of CBP by transcriptional activators/coactivators such as IRF-3, and E1A. Additionally, IBiD detects a variety of proteins, some of which lack a specific sequence association, while others are identified based on short structural motifs (89). Although the IBiD scaffold has been described through mapping the regions of p300 that bind to phosphorylated activation domain fragments from IRF-3, the minidomain can also bind to nonphosphorylated activation domain fragments of other proteins (90). Additionally, examination of the p300–p53 complex suggested the possibility that p300 binds to the N terminus of p53 *via* its IBiD domain (91).

### Intrinsically disordered regions

The extensive IDRs are dispersed throughout the p300/CBP sequence and are located mainly outside of the folded domains in the N- and C-terminal parts of p300/CBP flanking centrally located HAT-containing core (respectively, IDR1 to IDR5 between NRID and TAZ1, TAZ1 and KIX, KIX and BRD, TAZ2 and IBiD, IBiD and the C terminus). Besides their important regulatory role in modulating accessibility and enabling conformational flexibility of the protein, they facilitate interactions with various proteins (42, 43). IDRs were also shown to mediate the formation of dynamic membraneless transcriptional co-condensates or clusters with other transcription components, allowing for local high concentration of p300, promoting its efficient transactivation and amplifying gene expression in response to specific signals (57, 92).

## Regulation of p300/CBP

**Figure 2:**
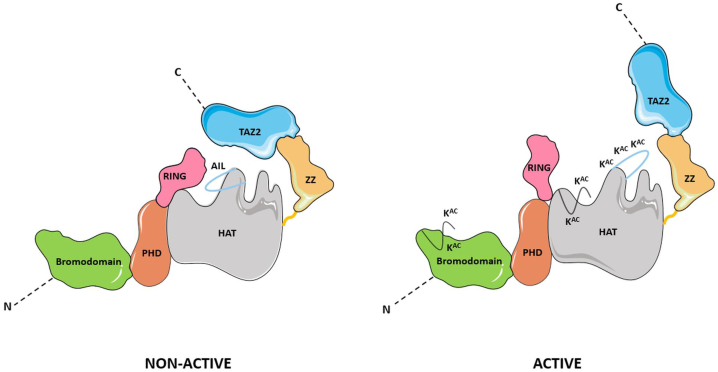
**Structural transition of p300/CBP from a nonactive to an active state.** In the nonactive conformation (*left*), the hypoacetylated AIL occupies the active site, thus preventing access of the potential substrate. Additionally, RING and TAZ2 domains sterically block the HAT catalytic pocket. In the active state (*right*), the hyperacetylated (KAc) AIL releases the active site obstruction, while repositioning of the RING and TAZ2 opens access to the catalytic pocket. AIL, autoinhibitory loop; CBP, CREB-binding protein; HAT, histone acetyltransferase; TAZ, transcriptional adapter zinc-binding domain.

The regulation of p300/CBP occurs at multiple cellular levels, primarily through intramolecular and intermolecular mechanisms with protein–protein interactions and PTMs playing a crucial role. Key mechanisms include p300/CBP self-regulation *via* the AIL, BRD, and PHD-RING region (Fig. 2), while interaction with various proteins modulates the p300/CBP activity, facilitating responses to various signaling pathways.

### Domain-dependent regulation of p300/CBP

Many of the p300/CBP domains are primarily associated with transcription factors, activators, and other components of multiprotein complexes involved in the regulation of a broad spectrum of proteins (see Table 1). Interestingly, the results of the latest research indicate the dominant role of the combinational binding of transcription factors to interacting domains, rather than the chromatin-interaction domains in specific p300/CBP recruitment to chromatin (93). Nonetheless, beyond being involved in the chromatin interaction, some of the p300/CBP domains have also been implicated in the direct or indirect self-regulation of p300/CBP activity (Fig. 2) and the recruitment of different protein targets through several adaptor domains is known to be an important regulatory mechanism of p300/CBP. These two acetyltransferases contains very conserved domains, including BRD, RING, PHD, and HAT that form the catalytic core (41, 94). These core domains are integral to the regulation of HAT activity through various interactions within the p300/CBP molecule itself as well as with other proteins (95). Consequently, the catalytic core not only directly drives p300’s enzymatic function but also serves as a site of regulation by various factors. The BRD, RING, and PHD regions, in particular, appear to be crucial in modulating HAT activity, highlighting their essential function in the overall regulation of p300/CBP (41).

#### Role of HAT and AIL

The AIL incorporated into the HAT domain plays an important role in the autoregulation of the p300/CBP catalytic activity that is governed by its acetylation status. The highly basic AIL domain, containing 13 lysine residues, facilitates an intermolecular process that enables trans-autoacetylation of up to 17 lysine residues within the AIL and surrounding area (96, 97). In its hypoacetylated form, the intrinsically disordered AIL competes with substrate binding, thereby inhibiting the catalytic activity of the HAT domain. However, once acetylated, the AIL undergoes a conformational change that releases the patch blocking the active site, thus promoting substrate binding and activating the HAT domain (85, 96). The autoacetylation of the lysine residues in the AIL is performed *in trans*, and other autoregulating mechanisms of p300 HAT activity involving the positioning of the RING domain prohibit *in cis* AIL autoacetylation (98). Moreover, the *in trans* AIL interaction is transient and does not result in dimer formation. Besides transautoacetylation of AIL, the inhibition of HAT can also be relieved by enhancer RNAs, which bind directly to the AIL and stimulate CBP acetyltransferase activity, promoting gene expression (99).

#### Role of BRD

BRD plays a critical role in regulating HAT activity. It is indispensable for effective substrate acetylation when functioning in conjunction with the HAT domain (41). The BRD may directly influence p300 HAT activity *via* intramolecular and/or intermolecular interactions with the AIL of p300, which leads to its hyperacetylation (98). Specifically, the BRD binds to the acetylated AIL, and it has been shown that such interaction in CBP is specific and requires acetylation of K1596. However, the BRD can also act as a negative regulator of histone acetylation, as it competes with the acetylated AIL for binding (100).

Although the absence of the BRD does not eliminate HAT activity, it significantly reduces transcriptional activity and substrate specificity (73, 100). Also, *in vitro* studies have demonstrated that the deletion of the p300 BRD impairs histone acetylation (101). This finding suggests that the BRD has a critical regulatory function in recruiting p300/CBP to hyperacetylated nucleosomes, thereby facilitating the initiation of p300/CBP-mediated acetylation (100).

#### Role of RING domain

Within the p300/CBP structure, the RING domain is positioned near the active site of the HAT domain, where it negatively affects the HAT activity by obstructing access to the substrate-binding cleft (95). The RING domain interacts directly with the substrate-binding loop of the HAT domain, forming a tight association. Deletion of the entire RING domain or mutations in residues that disrupt the RING–HAT interaction, or alter the structure of the RING domain, lead to an upregulation of HAT activity and consequently promote enhanced autoacetylation and acetylation of other substrates, such as p53 (41). Accordingly, mutations like R1645E and E1242K, which either disturb the RING–HAT interaction or remove the RING domain entirely, have been linked to various pathogenic conditions, including malignant melanoma and certain types of breast cancer (95).

The inhibitory effect of the RING domain on HAT activity is one of two key autoregulatory mechanisms for p300. In addition, p300 also possesses an innate capacity for self-activation by overcoming the RING domain’s inhibition through autoacetylation of the HAT domain. This process allows the nearby RING and PHDs to engage with potential substrates (41, 102).

In conclusion, the steric blockage of the HAT active site by the RING domain plays a critical role in the regulation of HAT activity. The RING domain needs to be repositioned for the HAT active site to be accessible to substrates, consequently, the RING domain seems to function as a gatekeeper. The complete catalytic activation that follows RING translocation is associated with autoacetylation *in trans* (41).

#### Role of the PHD

The PHD finger is characterized by a conserved Cys_4_-His-Cys_3_ motif, which coordinates two zinc ions, forming a compact structural unit essential for maintaining the integrity of the HAT domain in p300/CBP. Mutations that disrupt the zinc-coordinating residues within the PHD finger of CBP result in a complete loss of HAT activity. Correspondingly, disruption of zinc coordination through chelation leads to destabilization of the PHD finger, thereby impairing the activity of adjacent acetyltransferase domains (80). In contrast to CBP, the PHD finger of p300 does not appear to be required for HAT activity, indicating a domain-specific dependency (103). The BRD/PHD region is located in proximity to AIL and plays a role in regulating the self-acetylation of AIL by mediating interaction with the HAT domain in an acetylation-dependent manner. Notably, deletion of the BRD/PHD region resulted in a moderate reduction in HAT activity, whereas deletion of the PHD finger within this region led to a significant increase in p300 self-acetylation. These findings suggest that the PHD functions as a critical regulatory element modulating the acetylation status of the HAT domain and thereby influencing overall p300 functionality (81).

#### Role of TAZ domain

The distinct substrate specificities of TAZ1 and TAZ2 allow them to engage with different transcription factors, potentially influencing the recruitment of p300/CBP to chromatin loci (45, 104). TAZ2, in particular, serves as an E1A-binding ligand and interacts with the transactivation domains of several transcription factors, including p53. This interaction underscores TAZ2’s role in guiding p300/CBP to precise genomic locations, where it orchestrates key protein–protein interactions necessary for transcriptional regulation. Beyond its role in transcription factor recruitment, TAZ2 directly regulates HAT activity. Ibrahim *et al.* demonstrated that TAZ2 autoinhibited HAT by blocking the access of the substrate to the HAT active site with its C-terminal helix (amino acids 1806–1836). Interestingly, upon binding the ligand, TAZ2 undergoes a conformational rearrangement, releasing its inhibitory grip on HAT and thus facilitating its activation. This indicates that the recruitment of transcription factor and its connection *via* the activation domain with TAZ2 can activate HAT, highlighting the dynamic role of TAZ2 in modulating the enzymatic activity of p300/CBP (105). However, the role of TAZ2 as a negative regulator of HAT activity has also been suggested by the disruption of TAZ2, which was shown to enhance histone acetylation, particularly at H3K18 and H3K27 (106).

Additionally, emerging evidence indicated that the TAZ2 domain is essential for efficient acetylation of histone H3K27 by CBP *in vitro*. It has been shown that CBP associates with chromatin in a sequence-independent manner and that TAZ2 enhances CBP’s interaction with nucleosomes. This leads to an increase in CBP enzymatic activity and affects the change in the substrate preference. Notably, while the absence of TAZ2 does not impair CBP’s intrinsic enzymatic function, it alters substrate selectivity, highlighting its role as a modulatory interface within the p300/CBP complex (107).

#### Role of ZZ domain

The results obtained by Zhang *et al.* indicate that, beyond the BRD’s well-established role in acetyl lysine recognition, the ZZ domain of p300 is also critical for chromatin targeting and the catalytic activities of p300. While BRD is necessary for p300 to acetylate multiple residues in H3 and H4, the p300-ZZ provides the selectivity of the HAT domain toward the distal lysine sites in H3, such as H3K18 and H3K27 (67). Interestingly, however, this selective function appears to be unique to p300, as the CBP-ZZ domain does not exhibit the same specificity.

### Regulation of p300 at the transcriptional and posttranscriptional level

Chromatin remodeling *via* p300/CBP-dependent histone acetylation constitutes a pivotal and extensively characterized mechanism governing gene transcription. However, in contrast to its well-documented role in the regulation of other genes, the regulatory mechanisms governing p300/CBP at the transcriptional level remain relatively obscure. According to data from The Human Protein Atlas, the RNA expression profiles of both p300 and CBP exhibit minimal tissue and cell type specificity (https://www.proteinatlas.org/ENSG00000100393-EP300, https://www.proteinatlas.org/ENSG00000005339-CREBBP). Additionally, according to the Cancer Genome Atlas dataset, both p300 and CBP are prognostic markers in renal cancer. However, despite general similarity in tissue expression, detailed studies show some differences in the expression dynamics of the two acetyltransferases, particularly during development. p300 is often expressed earlier or more broadly, and CBP shows a more specialized role in certain cell types. During myoblast proliferation and differentiation, the mRNA and protein expression of CBP decrease, while that of p300 transiently increases (22). Similarly, a different pattern of spatial and temporal expression of mRNA and protein was observed for p300 and CBP during heart development, with earlier and more predominant expression of p300 (108).

Generally, the expression of p300 mRNA appears to be modulated by external stimuli depending on the signal and cell type. Yu *et al.* identified a zinc finger transcription factor—early growth response 1 (Egr1)—as a key mediator in the regulation of p300/CBP transcription in prostate cancer cells (109). The interplay between p300/CBP and EGR1 is intricate, involving multiple feedback loops that mediate transcriptional regulation. Depending on the cellular context, EGR1 may either induce or repress p300/CBP expression. Notably, EGR1-dependent p300/CBP upregulation in response to growth stimuli leads to the activation of downstream targets associated with cell survival and proliferation. However, it also results in the acetylation of EGR1 by p300/CBP, which provides negative feedback on its own, as well as p300/CBP transcription. Conversely, induction of EGR1 by UV-C irradiation stimulates its phosphorylation, thereby repressing p300/CBP transcription and contributing to apoptotic pathways (109).

Beyond EGR1, various additional factors have been implicated in the transcriptional regulation of p300/CBP across diverse cellular contexts. Bhattacharyya *et al.* identified the TGF-β–dependent enhancement of p300/CBP transcription in skin and lung fibroblasts (110). TGF-β stimulated p300, but not CBP expression at both mRNA and protein levels (111). Similarly, p300, but not CBP, is subject to transcriptional regulation by BRCA1 in breast cancer cell lines, wherein BRCA1 downregulates both p300 mRNA and protein expression (112). In these cell lines, BRCA1 downregulates p300 mRNA and protein. A diverse pattern of mRNA and protein expression of the two acetyltransferases was also observed in TET2-deficient cells. While the absence of TET2, an important epigenetic regulator in hemopoietic cells, significantly decreased p300 expression, it did not affect CBP (23).

Moreover, in human cells, mRNA and the protein expression of p300 and CBP can be induced by all-*trans* retinoic acid through retinoic acid receptors, transcriptional regulators interacting with p300 and CBP (113). Additionally, Tang *et al.* showed that cryptochrome circadian regulatory protein 2, involved in the pathway regulating bone marrow formation, negatively affects the expression of p300 by trapping CLOLK/BMAL1 transcription factor known to mediate p300 upregulation (114). While pSTAT1, rapidly activated by interferon gamma, binds and activates *EP300* in colon cancer (115).

The expression of *EP300* has also been shown to be regulated at the posttranscriptional level *via* miRNA. Sharma *et al.* reported that in a nonstressed cardiac myocyte, miR-142 suppresses *EP300* and p300-driven cytokine gene expression by binding to p300 3‘UTR (116). Reciprocally, under conditions of increased demand during cardiac myocyte growth, p300 leads to physiologic repression of miR-142. Furthermore, p300 is also a target of miR-132-3p, which decreases p300 protein expression and inhibits osteoblast differentiation (117). Additionally, miR-494-3p affects *EP300* expression by binding to its 3′-UTR during skeletal myogenesis (118).

### Regulation of p300 at the protein level

#### Regulation of p300 by PTMs

**Figure 3:**
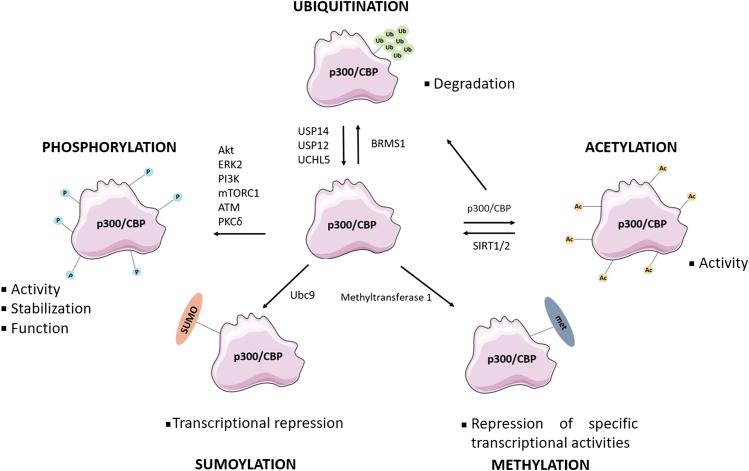
**Posttranslational modifications regulate the function, activity, specificity, and stability of p300/CBP.** While phosphorylation alters p300/CBP stability, activity, and function, acetylation improves its catalytic activity. On the other hand, sumoylation and methylation impose transcriptional repression through different mechanisms, while ubiquitination targets p300/CBP for degradation. CBP, CREB-binding protein.

p300/CBP protein is subject to various PTMs, including acetylation, ubiquitination, methylation, phosphorylation, and SUMOylation, which can impact the function and regulation of p300/CBP (Figure 3) (phosphosite plus, 2025).

##### Regulation of p300 by (auto)acetylation

Autoacetylation enhances the HAT activity of p300 by inducing a conformational change that results in the exposure of substrate-binding sites. This modification increases p300 affinity for histone substrates, thereby promoting transcriptional activation (96). Additionally, autoacetylation can prevent the association of p300 with repressive factors, ensuring its role in gene activation (97).

The AIL, located within the p300 HAT domain, serves as a pseudosubstrate and interacts with the HAT domain, blocking the substrate-binding site (96). Upon specific cellular signals, transcription factors dimerize and recruit p300 molecules into proximity. This spatial arrangement facilitates trans-autoacetylation, where one active p300 molecule acetylates the lysine residues present in the AIL domain of the other p300 molecule (98). Trans-autoacetylation causes a 4- to 10-fold increase in p300 catalytic activity (96). While autoacetylation converts the enzyme into its active conformation, the hypoacetylated state of AIL maintains the enzyme in an autoinhibited conformation, preventing substrate access to the active site (96).

Furthermore, p300 is a subject of deacetylation by sirtuins (SIRTs) (119). Among these, SIRT2 has been identified as the predominant p300 deacetylase, targeting acetylated lysine residues within the catalytic domain of p300 (120). In addition to SIRT2, SIRT1 interacts with p300 and selectively deacetylates two lysine residues (K1020 and K1024) within the cell cycle regulatory domain 1 (CRD1) motif, thereby attenuating p300’s transactivation potential (119). In line with this information, it has been shown that a sirtuin activator (resveratrol), indirectly affects the activity of p300 acetyltransferase, downregulating p300 protein levels by increasing p300 deacetylation and encouraging its ubiquitin-dependent degradation (121, 122).

##### Regulation of p300 by phosphorylation

Various kinases have been shown to modify p300/CBP protein by adding phosphate groups to multiple serine, threonine, and tyrosine residues. This PTM profoundly influences p300/CBP stability, enzymatic activity, and interactions with transcription factors. Depending on the context of signaling pathways and the specific residues modified, the phosphorylation may either enhance or suppress p300 function (123, 124). Remarkably, the phosphorylation level of p300 fluctuates dynamically throughout the cell cycle, underscoring its regulatory complexity (125).

Protein kinase B, also known as Akt, refers to a group of three serine/threonine-specific protein kinases that are integral to various physiological functions, including glucose metabolism, apoptosis, cell proliferation, transcription, and cell migration (126). Upon activation by upstream signals, Akt translocates to the nucleus, where it associates with p300 and phosphorylates it at specific serine residues, including S1834. This phosphorylation event enhances p300's intrinsic HAT activity, leading to increased acetylation of histones and transcription factors, thereby facilitating gene transcription (127). Additionally, Akt-mediated phosphorylation of p300 has been shown to influence its stability by preventing p300 degradation and ensuring its steady-state levels within the cell. This stabilization is crucial for sustaining the transcriptional regulatory functions of p300, thereby contributing to cell survival and proliferation (128).

Furthermore, the activation of extracellular signal–regulated kinase 2 mediates the phosphorylation of p300 C-terminal serine residues: S2279, S2315, and S2366, which stimulates the p300 HAT activity and controls p300 recruitment to the promoter of keratin 16 (129). CBP is also subjected to phosphorylation at S436 by the growth factor signaling pathways and at S301 modification by calmodulin kinase IV, both of which enhance CBP recruitment to transcriptional complexes and potentiate transcriptional activation (130, 131).

Moreover, mammalian target of rapamycin complex 1 directly interacts with p300 and phosphorylates it at four serine residues located at its C-terminus: S2271, S2279, S2291, and S2315. This phosphorylation disrupts the interaction between the HAT and RING domains of p300, thereby potentiating its acetyltransferase activity. Functionally, mammalian target of rapamycin complex 1–dependent phosphorylation of p300 suppresses autophagy and plays a pivotal role in cellular metabolism by regulating anabolic and catabolic processes (132).

A serine–threonine kinase, ataxia-telangiectasia mutated, phosphorylates p300 at S106 upon DNA damage. S106A mutation in p300 in response to DNA damage leads to the instability of both p300 and a DNA repair protein, NBS1, involved in homologous and nonhomologous recombination (133).

In contrast to other phosphorylation sites detected in CBP/p300, the phosphorylation at S89, performed by the isoform δ of a signal-dependent PKCδ, specifically reduces the transcriptional activity of p300 (134, 135).

##### Regulation of p300 by ubiquitination

The activity of p300 is intricately regulated by PTMs, among which ubiquitination serves as a pivotal determinant of its stability, function, subcellular localization, and interactions with regulatory proteins. The level of p300 protein is mainly regulated by polyubiquitination, which directs the protein to degradation by the 26S proteasome (136, 137, 138, 139, 140).

The ubiquitination of p300 is orchestrated by E3 ubiquitin ligases, which recognize and facilitate the transfer of ubiquitin from E2 conjugating enzymes to p300 (95). Liu *et al.* identified that breast cancer metastasis suppressor 1 (BRMS1) functions as a p300 E3 ligase of p300 and polyubiquitinates it, resulting in proteasome-mediated degradation. Interestingly, BRMS1 binds to the C-terminal part of p300 (region between 1905 and 2414 aa); however, the specific lysine residues subjected to polyubiquitination by BRMS1 appear to be located in different regions of the acetyltransferase (139). Moreover, it has been shown in lung cancer cells that the ubiquitin/proteasome-dependent degradation of p300 is enhanced by the cyclic AMP signaling system, which consequently reduces the levels of p300 protein (140).

Ubiquitination is a reversible process, and some deubiquitinases that can remove ubiquitin chains from p300 and consequently stabilize its protein have been identified. Ubiquitin-specific protease 14 and ubiquitin C-terminal hydrolase L5 have been shown to interact with p300, deubiquitinate and stabilize it, which in turn may promote renal fibrosis. Conversely, the inhibition of these deubiquitinases by the specific inhibitor bAP-15 led to increased ubiquitination and subsequent degradation of p300, resulting in the suppression of fibrotic processes (141). Another deubiquitinating enzyme, ubiquitin-specific protease 12, has also been shown to remove ubiquitin chains from p300, preventing its proteasomal degradation (142).

Interestingly, a form of competition between ubiquitination and acetylation of p300 has been observed. Li *et al.* reported induction of p300 degradation in response to histone deacetylases (HDACs) inhibition, while Chen *et al.* indicated that powerful inhibitors of HDAC, such as valproic acid or butyrate, induce redistribution of p300 to the cytoplasm and its degradation through the 26S proteasome pathway (143, 144). Inhibition of HDACs leads to histone hyperacetylation, transcription activation, and a potential lesser requirement for p300. Thus, the removal of p300 from the nucleus may serve to control its availability and functioning by limiting the opportunity to interact with transcription regulators in response to particular cellular conditions. Interestingly, shuttling of p300 to cytoplasmic inclusion bodies depends on microtubules and involves ubiquitination of p300. Additionally, Kuno *et al.* demonstrated in cardiomyocytes that SIRT1 deacetylates p300, thereby stimulating its ubiquitination and degradation (122).

##### Regulation of p300 by sumoylation

Covalent attachment of small ubiquitin-like modifier (SUMO) to lysine residues can also affect p300 functionality and regulate its transcriptional responses (145, 146). Girdwood *et al.* have identified the CRD1 motif in p300 (aa 1017–1029), which encompasses two copies of the sequence ψKxE (where ψ is a large hydrophobic amino acid and x I any amino acid), recognized as a consensus for modification by SUMO-1. Sumoylation at the CRD1 motif, most likely facilitated by the SUMO conjugation enzyme UBC9, has been shown to mediate transcriptional repression (145). Respectively, Park *et al.* presented that BRD, PHD, and ZZ domains of CBP function as an E3-SUMO ligase and together with UBC9 promote intramolecular sumoylation of the CRD1 domain, which is the most efficient in *cis* (100). Furthermore, it has been reported that the ZZ domain of p300/CBP can interact with SUMO-1 (147).

These findings indicate that p300/CBP can function as either a transcriptional activator or a repressor in response to distinct cellular stimuli, depending on the lysine modifications, specifically, acetylation *versus* sumoylation.

##### Regulation of p300 by methylation

p300/CBP also undergoes arginine methylation. Even though there is no apparent effect on acetyltransferase activity, it significantly modulates p300/CBP transcriptional coactivator functions (148, 149). This modification is facilitated by the coactivator-associated arginine methyltransferase 1 (also referred to as PRMT4), which targets and conserves arginine residues located within the KIX domain. Notably, R580 in p300, and analogous R600 conserved in CBP, are the most highly methylated residues in p300/CBP KIX domains. The methylation of those sites hinders the ability of p300/CBP to interact with CREB *via* the KIX domain and consequently blocks CREB activation (149). Alternatively, Chevillard-Briet *et al.* pointed to three conserved arginine residues (R714, R742, and R768 in CBP, and respective arginine residues in p300), located at the edge of the KIX domain, as the major methylation sites for CARMI *in vitro* (148). It has been indicated that CBP methylation is required for GRIP-1–induced and steroid hormone-induced gene activation. Consequently, the methylation of p300/CBP by coactivator-associated arginine methyltransferase 1 intricately regulates their coactivator functions, selectively modifying interaction dynamics and influencing gene expression responses.

#### Regulation of the p300 by protein degradation

Multiple independent reports showed that the p300 protein can be degraded in ubiquitin-dependent proteolysis (136, 137, 138, 139, 140).

BRMS1 is one of the E3 ubiquitin ligases that polyubiquitinate p300 (139). A recent study identified yet another E3 ubiquitin ligase, TRIM25, which affects proteasome-dependent degradation of p300 without its direct polyubiquitination. TRIM25 was shown to promote the interaction of p300 with a motor protein, dynein, which moves along microtubules, transporting intracellular cargo, and consequently facilitated the transport of p300 to the proteasome (150).

#### Regulation of p300 by protein–protein interactions

The regulation of p300/CBP by protein–protein interactions plays a crucial role in its ability to integrate signals from multiple cellular pathways. *Via* multiple protein-interacting domains, p300/CBP can bind a wide range of proteins, including transcription factors, chromatin modifiers, and other regulatory proteins (see table 1). Some interacting proteins directly bind to DNA, often in response to specific signals, and recruit p300/CBP to target gene elements, facilitating transcriptional regulation. One of the mechanisms involves the competition for binding sites within particular domains, such as the CH3 domain, which hosts numerous potential protein partners that compete for the same recognition sequences within p300/CBP. Such competition helps modulate the function and specificity of p300/CBP in various signaling contexts (151). One of the examples is a transcriptional repressor C-terminal binding protein, which interacts directly with the p300 BRD and affects the interaction between the BRD and histones in an NADH-sensitive manner, hindering the accessibility of p300 to histones (71). The acetyltransferase activity of p300/CBP is also a strong inhibitor target of the adenoviral E1A oncoprotein. E1A can interact with the HAT domain directly and reduce p300 acetyltransferase activity *in vitro* (152).

Additionally, some p300 interacting partners are involved in the positive regulatory loops of acetyltransferase activity. Sen *et al.* showed that p300/CBP interacts with nuclear GAPDH and acetylates it at K160. In turn, the direct interaction stimulates the catalytic activity of p300/CBP (153). Mastermind-like protein-1 (MAML1) can directly interact with the CH3 domain of p300, and the p300–MAML1 complex has been shown to preferentially acetylate the tails of histones H3 and H4 in chromatin *in vitro* (154). However, MAML1 can induce p300 autoacetylation, resulting in the enhancement of p300 HAT activity (155). In an aggressive cancer, nuclear protein in testis (NUT) carcinoma, a testis-specific factor, NUT forms a fusion with *BRD4*. The BRD4-NUT fusion protein binds p300 *via* its TAZ2 domain and induces p300 activity 8 to 10 times (156). Moreover, Ibrahim *et al.* indicated that the BRD4-NUT system activates p300 by affecting an autoinhibitory function of the TAZ2 domain (105).

## Association of p300/CBP with diseases

Serious disorders have been linked to the dysfunction or misregulation of p300/CBP. The loss-of-function mutations affecting acetyltransferase activity lead to severe diseases, including RSTS, MHS, and cancer [(33, 82), reviewed in (35)]. However, gain of function in p300/CBP, by direct mutations or overexpression of p300/CBP, also has harmful consequences, underlining thus the importance of p300/CBP correct regulation and maintaining the balance between acetylation and deacetylation. One such example is an implication of p300/CBP in the pathogenesis of multiorgan fibrosis (157).

### Rubinstein–Taybi syndrome

Discovered in 1963, the RSTS is inherited as an autosomal dominant trait, but generally caused by *de novo* mutations in one of two genes, *CREBBP* or *EP300*, and appears with a frequency of 1:100,000 (158, 159). However, RSTS caused by mutations in *CREBBP* is more common (55–75%) and has a more severe phenotype than the ones located in the *EP300* gene (identified in 8–11% of individuals with RSTS) (30, 160). The syndrome is a multiple systems disorder characterized by dysmorphology including facial features, broad thumbs and large toes (158), abnormal distal phalanges, several organ anomalies (161, 162, 163, 164), elevated cancer risk, and mental retardation (165, 166, 167). *CREBBP* mutations that cause RSTS can range in size from very extensive deletions that eliminate hundreds of kilobases to deletions that only affect one exon [reviewed in (159)]. Since the *CREBBP* and *EP300* genes encode HATs, RSTS is believed to arise from defective histone acetylation, leading to transcriptional dysregulation (168). Interestingly, the analysis of RSTS etiology also pointed to a single amino acid change (R1379P) in the PHD of CBP as a cause of a significant decrease in the HAT activity (169). It indicated that the integrity of the PHD-type zinc finger in the HAT domain can affect the enzymatic activity of CBP and cause RSTS. In support of this assumption, the study carried out by Kalkhoven *et al.* showed that, besides eight unique heterozygous variants in the HAT domain of CBP found by mutational analysis in 39 RSTS patients, one (E1278K) was located in the PHD finger, while the other mutation removed exon 22, encoding the core part of the PHD finger (82).

### Menke–Hennekam syndrome

MHS is a *de novo*, congenital, autosomal dominant disorder. Its most frequently described characteristics include intellectual deficiencies, autistic behavior, reduced motor skills, feeding difficulties, facial dysmorphism, and other structural features, including a petite physique and microcephaly (33). MHS is caused by mutations in 30 and 31 exons of *CREBBP* (MHS type I) or exon 30 of *EP300* (MHS type II) (170, 171, 172, 173, 174). In addition to exon 30 and 31 mutations, pathogenic variants in the ZZ and TAZ2 zinc-finger domains and the fourth intrinsically disordered linker (ID4) have also been implicated in MHS (175). The ZZ and TAZ2 domains are critical for mediating protein–protein interactions with other transcriptional regulators and are essential for the recruitment of transcriptional coactivators (45, 66, 105). Mutations in these domains might disrupt normal protein function, leading to the abnormalities observed in MHS.

A recent study has claimed that there are three MHS subtypes based on the specific domains affected: MHS-ZZ, MHS-TAZ2, and MHS-ID4. Each subtype exhibits distinct phenotypic characteristics, including varying degrees of intellectual disability and unique physical features (175).

### Cancer

Both p300/CBP dysfunction, as well as its overproduction, have been associated with various cancers, including melanoma, hepatocellular carcinoma, sarcoma, glioblastoma, leukemia, and lymphoma, as well as breast, prostate, kidney, and lung (recently reviewed by (176). Mutations in *CREBBP* and *EP300* are connected, among others, with multiple hematological malignancies. It has been shown that chromosome translocations affecting the *CREBBP* relate to a subtype of acute myeloid leukemia (177, 178). Additionally, *CREBBP* and *EP300* variants have been observed in aggressive NK cell leukemia (179) and childhood acute lymphoblastic leukemia (180). Furthermore, *CREBBP* and *EP300* mutations affecting acetyltransferase activity are the most frequent cause of diffuse large B-cell lymphoma (181, 182).

Beyond hematological malignancies, p300/CBP alterations have been linked to a broad spectrum of solid tumors. Somatic mutations in the genes encoding p300/CBP were found in small cell lung cancer (183), colon cancer (184), colorectal cancer (185), esophageal cancer (186), breast cancer (187, 188), gastric cancer (189), and bladder cancer (190).

Overexpression of p300/CBP also correlates with cell transformation. Such examples have been found in many solid tumors, including colorectal, prostate, lung, and breast cancer, and they have been related to malignancy, tumor progression, a reduced rate of longevity, and poor prognosis (191, 192). Additionally, upregulation of p300 was pointed out as a significant factor in the hepatocellular carcinoma tumorigenic process, contributing to its enhanced malignant properties and/or poorer prognosis (193, 194).

Interestingly, some cancers are related to dysfunction of other p300/CBP domains than HAT. Deletions of residues encompassing BRD were found in cervical cancer (28). Additionally, mutations in the R1645 residue, which disrupt interactions between the RING domain and the active site, are implicated in malignant melanoma (95).

Mutations in the p300 gene can also affect metastatic invasion. The R397Q mutation in p300, absent in normal tissues and primary tumors, has been detected exclusively in metastatic lung tumors, suggesting that p300 mutations may drive tumor spread (195).

Although p300 was described as a tumor suppressor in several cancer types, including breast, colorectal, and gastric carcinoma, it has also been found to act as a positive regulator of cancer progression and is associated with the tumorigenesis of several human cancers (193).

## Regulation of p300/CBP in therapies

Overexpression or dysregulation of p300/CBP can lead to tumorigenesis, but it has also been related to other disorders such as hypertrophy, multiorgan fibrosis, or diabetes, and the role of p300/CBP in viral transformation has been shown (reviewed in (3)). It indicates a potential therapeutic value of p300/CBP, and over the years, multiple p300/CBP-targeted inhibitors have been identified and tested. The p300/CBP inhibitors, based on their target, can be categorized into different groups. Most of the inhibitors aim to block the p300/CBP HAT catalytic domain and compete with acetyl-CoA binding (*e.g.*, A-485, C646) (4, 196, 197). Others, such as SGC-CBP30 or I-CBP112, target BRDs and block their interaction with acetylated proteins, effectively preventing p300/CBP from accessing its substrate on chromatin (78, 198). Additionally, bisubstrate mimic inhibitors are designed to have two parts: one fitting into the acetyl-CoA binding pocket and the other resembling the peptide substrate, thereby mimicking the enzyme’s natural substrates (199). Another type of inhibitors, proteolysis-targeted chimera, are bifunctional molecules that simultaneously bind an E3 ubiquitin ligase and a protein of interest, inducing degradation of the latter *via* the ubiquitin-proteasome system, gaining more interest as potential therapeutics targeted to p300/CBP (200, 201). As well as rationally designed compounds including synthetic small-molecule inhibitors, there are naturally derived products, such as garcinol, curcumin, plumbagin anarcadic acid that are known p300/CBP inhibitors (202, 203, 204, 205).

One also needs to remember that, besides modifying proteins by acetylation, p300/CBP also functions as a scaffold for numerous proteins, and this role can be affected indirectly by inhibitors.

According to ClinicalTrials.gov, several p300/CBP inhibitors are currently undergoing clinical trials. The first p300/CBP inhibitor, which entered the clinical trials, CCS1477, is being evaluated in a phase I/IIa clinical trial for patients with advanced solid tumors, including metastatic breast cancer, non–small cell lung cancer, and metastatic castration-resistant prostate cancer. The safety and efficacy of CCS1477 have already been assessed in an open-label phase I/IIa study as a monotherapy for patients with advanced solid or metastatic tumors (ClinicalTrials.gov ID: NCT03568656). Another p300/CBP inhibitor, EP31670, is currently in a phase I first-in-human study targeting advanced solid tumors and hematologic malignancies (ClinicalTrials.gov ID: NCT05488548). Additionally, a phase I open-label study was conducted to assess the safety and tolerability of the p300/CBP inhibitor FT-7051 in men with metastatic castration-resistant prostate cancer (ClinicalTrials.gov ID: NCT04575766). All three inhibitors mentioned above target the BRD. Furthermore, a recent dose-finding study is underway to evaluate the safety and preliminary antitumor activity of the p300/CBP BRD inhibitor pocenbrodib, either as a monotherapy or in combination with abiraterone acetate, olaparib, or 177Lu-PSMA-617, in patients with metastatic castration-resistant prostate cancer (ClinicalTrials.gov ID: NCT06785636).

## Conclusions and perspectives

The regulation of p300/CBP acetyltransferase is orchestrated by a sophisticated and intricate network of molecular mechanisms that collectively determine its activity, stability, and functional specificity. The process involves multiple layers of control, including protein–protein interactions, PTMs, translational control, protein degradation, transcriptional, and posttranscriptional regulation. The interplay among these regulatory pathways allows for precise modulation of p300/CBP function in response to cellular and environmental signals, underscoring its essential role in maintaining cellular homeostasis. Understanding how these regulatory mechanisms coordinate in a timely manner, especially given the broad substrate specificity and cellular context, remains an intriguing and ongoing area of research.

Due to the substantial general sequence similarity, and particularly a high identity of catalytic domains, p300 and CBP are often considered to be interchangeable. However, some distinct functions, underlined by inability to substitute one protein by the other, and some more or less subtle variances in regulation, state otherwise. Distinguishing the molecular role, as well as identifying independent inhibitors of p300 and CBP, is challenging. However, an in-depth analysis of the regions with lower similarity, such as the NRID or IDRs might be crucial for understanding both acetyltransferases’ unique contributions and for developing specific inhibitors.

Depending on binding and cell type, p300 can act not only as a tumor suppressor but also as a tumor promoter. In cancers of the lung, stomach, pancreas, and prostate, p300 has been shown to facilitate malignant progression [reviewed (176, 206)]. In contrast, in epithelial-origin cancers, p300 often exhibits tumor-suppressive properties (12). It indicates that p300 activity, beneficial in one type of tissue, may contribute to disease progression in another type. Such duality underlines the necessity of tailoring treatments targeting p300 activity in tissue- and cancer-dependent manner.

Given its essential role in controlling many cellular processes, either through interaction with over a hundred partners or by affecting directly or indirectly the expression of multiple genes, small perturbations in the regulation of p300 can have widespread biological consequences.

Playing a critical function in chromatin remodeling, transcriptional regulation, and signal transduction, p300 has emerged as a compelling target for therapeutic intervention. The development of small-molecule inhibitors and other pharmacological modulators of p300/CBP function hold promise for the treatment of cancer and other p300-associated disorders.

Future investigations should aim to unravel the context-dependent regulatory mechanisms of p300, exploring how distinct signaling pathways converge to modulate its activity in different physiological and pathological states. Moreover, advances in epigenetic therapies and targeted modulation of acetyltransferase activity may offer new avenues for clinical intervention.

## Conflict of interest

The authors declare that they have no conflicts of interest with the contents of this article.
